# Abstract of the Proceedings of the Cook County Medical Society

**Published:** 1853-07

**Authors:** De Laskie Miller

**Affiliations:** The Secretary


*Abstract of the Proceedings of Cook County Medical Society, at a Meeting held July 5th*, 1853. By the Secretary, DeLaskie Miller, M.D.

Dr. Morfit read a paper, directing the attention of the members of the society to the effects of the vapor of mercury and bromine upon the system, as experienced by persons engaged in the daguerrean art, such as suppuration of the lymphatic glands, especially of the inguinal region, and abscesses in the axilla.

Dr. Bloodgood remarked, that he had seen some accounts of the effects of bromine upon the system, especially upon the respiratory organs, but thought it somewhat questionable, whether the vapor of mercury caused the abscesses described by the paper. Physicians are constantly in the habit of prescribing mercurials to discuss inflammation of lymphatic glands.

Dr. Freer was of the opinion that it is quite probable, that the fumes of mercury may cause inflammation of the glands, like that referred to in the paper; for it is a matter of common observation, that the continued use of mercury, causes great irritation of the glandular system. He considered the subject one of interest, and moved, that the discussion be continued at the next meeting of the society.

An interesting paper was read by Dr. Freer, describing the experiments made by Dr. Marshall Hall, during his recent visit to this city.

Dr. Palmer said, that in order to fully understand and appreciate these experiments, it is necessary to have some precise ideas of Dr. Hall's theory, and also of Dr. Dowler's theory and experiments upon the alligator, upon which it was founded; and suggested that some of the members who had recently been paying attention to the subject, should give a precise statement of the points of difference between Dr. Hall and Dr. Dowler.

Herrick explained, that Dr. Dowler assumes that there is a diffuse sensorium, and that sensation pervades every part of the system, independent of the encephalon. Dr. Hall contends that his experiments demonstrate, that through the connection of the reflex system of nerves with the spinal cord, the power of motion remains after the brain has been severed, but, that sensation and volition are lost.

Dr. Johnson remarked, that there is a question of fact between Dr. Dowler and Dr. Hall. Dr. Dowler asserts, that after the removal of the encephalon and destruction of the spinal cord, there was still motion, apparently voluntary and directed by intelligence. Dr. Hall claims, and his experiments prove, (as Dr. J. thinks) that after the destruction of the brocere-spinal axis, there are no motions either voluntary or involuntary.

Dr. Palmer inquired, if Dr. Hall had ever experimented upon the alligator, and if he was a proper judge of Dr. Dowler's facts? and suggested that there was a difference in organization between that animal and a frog.

Dr. Davis said, he had bestowed some thought upon the subject, and had a remark to make. Thinks it questionable, whether we are ever to arrive at any satifactory conclusions upon this subject from this manner of experimenting. Experiments like those referred to, made upon animals of different species and of a different organization, to establish the truth of one hypothesis and disprove another, can lead to nothing but confusion. We must go back to what is no new doctrine in the field of physiological inquiry, and that is this, that there is a gradation in animal organization, causing a wide difference in the nature and habits of different species, and therefore it is but reasonable to conclude, that experiments made upon different species of animals to elucidate a peculiar hypothesis, must lead to different conclusions.

Dr. Johnson read a paper on "Oxalate of Lime and its relations to certain forms of Neuralgia," in which he expressed the opinion, that they often sustain to each other an intimate relation through a common cause, viz: a derangement of the digestive organs. He was led to this conclusion from the fact that in a large number of instances in which he had discovered oxalate of lime in the urine, the patients were affected not only with dyspepsia, but were subject to frequent neuralgic attacks, the severity of which, bore a direct relation to the quantity of the oxalic deposit.

He also called the attention of the society to the influence of citric acid, in producing oxalic deposits, and related some experiments, which seemed to prove, that the use of orange, currants, whortleberry, and perhaps other fruits containing citric acid, favor the production of oxalic acid.

The discusssion of this paper was deferred till the next meeting of the society.

---

For the North-Western Med. and Surg. Journal.

MESSRS. EDITORS,

The following Circular in relation to the next volume of Transactions of the American Medical Association was received a few days since, and I know of no better way to call the attention of the profession, in this section of country, to it, than to procure its insertion in the columns of your journal. From what I know of the contents, I can assure your readers that the *Sixth*, or forth-coming volume, will be one of great value ; one of the Essays, with its illustrations being alone worth more than the *five* dollars required for the whole. I trust, therefore, that many of your readers will secure for themselves a copy by remitting early the required amount. The following is the Circular, viz:

PHILADELPHIA, June 14, 1853.

DEAR SIR,

In conformity with a resolution of the American Medical Association, it becomes my duty to inform you of the decision of the Committee of Publication in regard to the price at which the forthcoming volume of the Transactions will be furnished to the members of the Association and to others.

The price of *single copies* has been fixed by the Association at *five dollars*.

Any *individual* desiring two copies, will be furnished with them upon his remitting to the Treasurer *nine dollars*.

Societies, Associations, and Institutions requiring *six* copies for the use of their members, will be supplied with them on remitting to the Treasurer *twenty-five dollars*, or they will be supplied with twenty-five copies on remitting *seventy-five dollars*.

Those who wish volumes 5 and 6, may obtain them by remitting *eight dollars*.

In consequence of the numerous illustrations, many of them highly finished colored lithographs, with which the forthcoming volume will be accompanied, its cost will considerably exceed that of either of those previously issued by the Association. To defray the cost of its publication, at least *fifteen hundred dollars*, in addition to the amount already received by the Treasurer, will be required.

Your attention is called to the following resolution adopted at the last session of the Association.

"*Resolved*, That the Delegates from the several States be requested to appoint Committees, who shall aid the Committee of Publication in procuring subscribers for, and in distributing the Annual Transactions of the Association."

Respectfully, yours,

D. FRANCIS CONDIE,
*Treasurer, and Chairman Com. of Pub. A. M. A.*

Very respectfully yours,

N. S. DAVIS.

Chicago, July, 1820.

We cheerfully give place to the above Circular and Note from Prof. DAVIS, especially as we fear that the Publishing Committee have forgotten that there is such a work as the *North Western Medical and Surgical Journal.*

We should be very glad to look over the last volume. What say you, Dr. CONDIE; shall we have the pleasure of expressing our opinion on any of its contents?—(EDS. N. W. MED. & SURG. JOURNAL.)

